# Very Low-Carbohydrate High-Fat Diet Improves Risk Markers for Cardiometabolic Health More Than Exercise in Men and Women With Overfat Constitution: Secondary Analysis of a Randomized Controlled Clinical Trial

**DOI:** 10.3389/fnut.2022.867690

**Published:** 2022-05-23

**Authors:** Lukas Cipryan, Martina Litschmannova, Philip B. Maffetone, Daniel J. Plews, Tomas Dostal, Peter Hofmann, Paul B. Laursen

**Affiliations:** ^1^Department of Human Movement Studies & Human Motion Diagnostic Centre, The University of Ostrava, Ostrava, Czechia; ^2^Department of Applied Mathematics, VSB—Technical University of Ostrava, Ostrava, Czechia; ^3^Independent Researcher, Brattleboro, VT, United States; ^4^Sports Performance Research Institute New Zealand (SPRINZ), Auckland University of Technology, Auckland, New Zealand; ^5^Institute of Human Movement Science, Sport & Health, Exercise Physiology, Training & Training Therapy Research Group, University of Graz, Graz, Austria

**Keywords:** HOMA-IR, adiponectin, leptin, TG/HDL-C, TyG index, low carbohydrate diet, exercise, overfat

## Abstract

**Purpose:**

This randomized controlled parallel-group study examined the effects of a very low-carbohydrate high-fat (VLCHF) diet and high-intensity interval training (HIIT) program over 12-weeks on cardiometabolic risk factors in individuals with overfat constitution.

**Methods:**

Ninety-one participants out of 109 completed the study. The participants were randomly allocated to the HIIT (*N* = 22), VLCHF (*N* = 25), VLCHF+HIIT (*N* = 25), or control (*N* = 19) groups for 12 weeks. Fasting plasma samples were collected before the intervention and after 4 and 12 weeks. The analyzed outcomes included complete blood count, glucose, insulin, glycated hemoglobin, triglycerides (TG), cholesterol, high- and low-density lipoprotein (HDL-C and LDL-C), lipoprotein(a), adiponectin (Adpn), leptin (Lep), tumor necrosis factor α (TNF-α), other interleukins (hs-IL-6, IL-1β, and IL-10), and IL-1RA. The homeostasis model assessment of insulin resistance (HOMA-IR), Adpn/Lep ratio, TG/HDL-C ratio, and TyG index were calculated and analyzed. Blood pressure was measured before the intervention, after 4, 8, and 12 weeks (ClinicalTrials.gov: NCT03934476).

**Results:**

Absolute changes in HOMA-IR, Adpn/Lep ratio, LDL-C, and diastolic blood pressure after 12 weeks differed by study groups (*p* < 0.05). The most pronounced changes were revealed in the VLCHF (ΔM [95% CI]; HOMA-IR: −0.75 [−1.13; −0.55]; Adpn/Lep: 9.34 [6.33; 37.39]; LDL-C: 0.06 [−0.12; 0.50] mmol/l) and VLCHF+HIIT (HOMA-IR: −0.44 [−1.14; 0.12]; Adpn/Lep: 4.26 [2.24; 13.16]; LDL-C: 0.25 [−0.04; 0.50] mmol/l) groups.

**Conclusions:**

A 12-week VLCHF diet intervention in individuals with overfat constitution is effective for favorable changes in HOMA-IR (compared to HIIT), Adpn/Lep ratio, and diastolic blood pressure. HIIT, or HIIT combined with the VLCHF diet, had no additional benefits for the analyzed variables. No adverse side effects were observed.

## Introduction

Physical activity levels in the Western population have not reduced and changed little, despite dramatic increases in the overfat pandemic of the past 30-plus years. More than half of US adults meet the federal 2008 *Physical Activity Guidelines for Americans* and regular exercise using either aerobic or muscle-strengthening exercise, increasing from 44% in 1998 to almost 52% in 2014 (1). Those meeting these guidelines for aerobic activity and muscle-strengthening exercise also increased from about 14% in 1998 to 21% in 2014. However, rates of adults with overweight or obesity rose to almost 71% during a similar period, reflecting the overfat prevalence increase from 75% to over 90% (2).

Cardiovascular and metabolic (cardiometabolic) risk factors can contribute significantly to increased morbidity and mortality, reduced quality of life, and higher healthcare costs. Three of the major risk factors include excess body fat, low-grade systemic chronic inflammation, and insulin resistance (IR). *Overfat* is defined as excess body fat that impairs health (3). Determination of overweight and obese classifications are traditionally based on measures of body mass index (BMI). This is not a direct measure of body fat and can misclassify up to 50 % or more patients with both increased body fat and its associated disease risk factors. Therefore, body fat needs to be considered directly to assess a high-risk body composition (4).

Overfat, and its downstream IR and chronic inflammation, which can maintain a diet-induced viscous cycle, can also lead to a wide range of cardiometabolic health problems such as metabolic syndrome, atherosclerosis, hypertension, dyslipidemia, and advanced chronic conditions such as Type 2 diabetes, cardiovascular diseases, cancers, and neurodegenerative diseases (2). In addition, fasting triglycerides and high-density lipoprotein cholesterol, particularly the ratio of these two measures (TG/HDL-C), are also considered a significant cardiometabolic risk factor as the ratio reflects IR (5). The TG/HDL-C ratio may be a better clinical screening index than the homeostasis model assessment of IR (HOMA-IR) due to accessibility, reproducibility, and cost, and is already a commonly used measure in clinical practice.

Exercise and diet are two commonly used modifiable lifestyle factors that can help reduce cardiometabolic risk factors to influence morbidity and mortality, improve quality of life, and reduce healthcare costs. While the importance of physical activity for increased fitness is undeniable, exercise alone may not necessarily reduce excess body fat. In a previous randomized controlled clinical trial we showed that a very low-carbohydrate high-fat (VLCHF) diet alone reduced excess body fat in individuals with overfat constitution more than high-intensity interval training (HIIT) alone (6). Low carbohydrate diets are effective in remission of diabetes (7) and improve insulin sensitivity as measured by HOMA-IR (8–10). Similarly, insulin sensitivity is also related to the degree of physical activity. Exercise has been shown to ameliorate insulin action in insulin-resistant individuals (11) by improvement of the pathophysiologic pathways involved in insulin resistance (12). The beneficial exercise effect on insulin sensitivity occurs after several weeks (13) or even after a single bout of exercise in adults with obesity (14). Moreover, it seems that HIIT induces similar acute improvements in peripheral insulin sensitivity as moderate-intensity continuous training (15).

A VLCHF diet was previously shown to increase adiponectin/leptin ratio reflecting reduced systemic low-grade inflammation in healthy young individuals (16). In contrast, the same diet was found to be an effective strategy for reducing excess body fat in men and women with overfat constitution (6). Systematic reviews and meta-analyses show also beneficial effects of low carbohydrate diets combined with exercise on body composition, triglycerides, and aerobic capacity in adults with obesity (17, 18). Consuming a high fat diet, especially one with high saturated fatty acids (SFA), is thought to impair key aspects of cardiometabolic health. This is despite no evidence-based associations between high intake of SFA and risk of atherosclerotic progression (19). Therefore, the purpose of this randomized controlled parallel-group study is to examine the effects of a 12-week VLCHF diet and high-intensity interval training (HIIT) program on cardiometabolic risk factors in men and women with overfat constitution aged 20–59 years.

## Methods

### Parent Study

**Figure 1:**
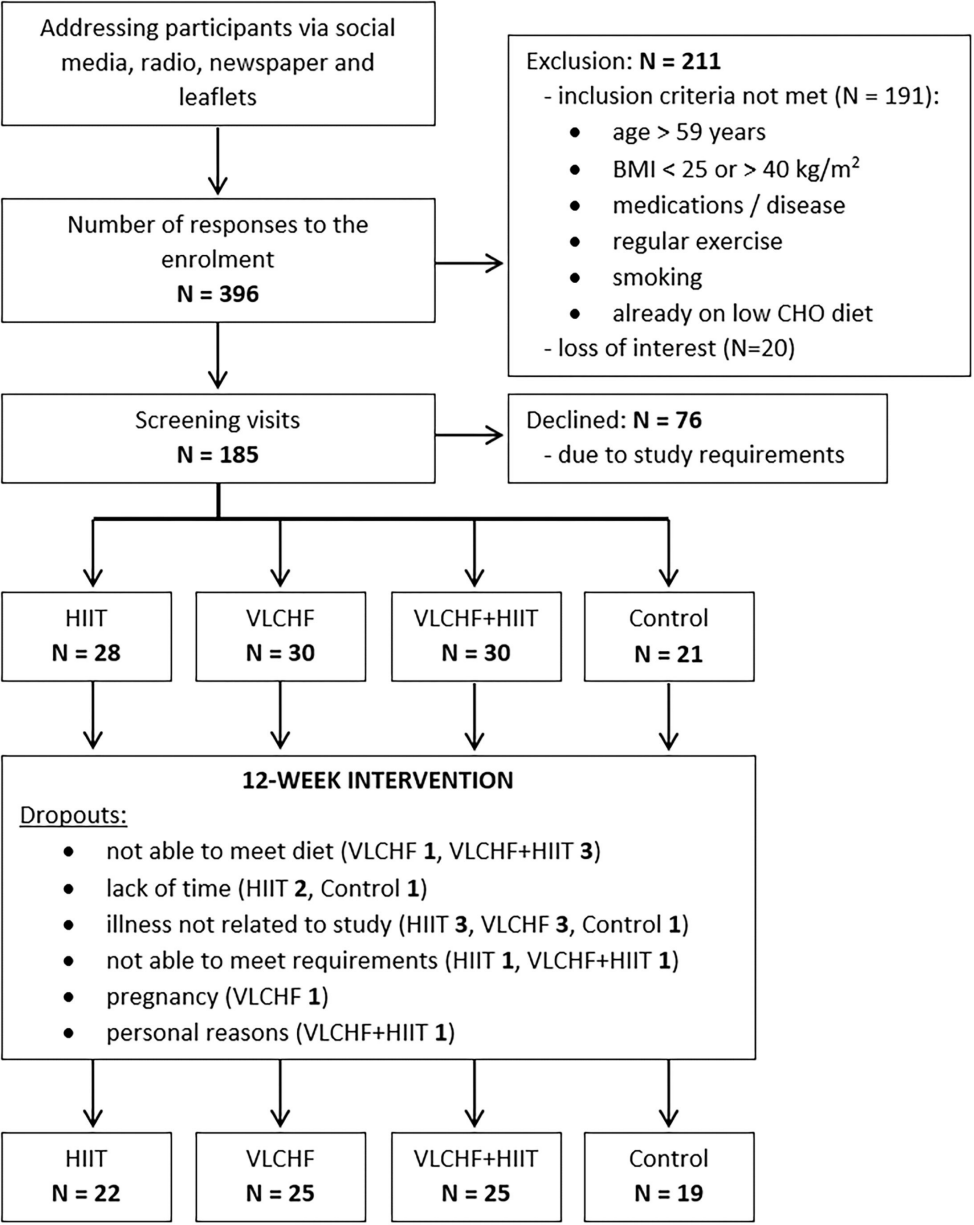
Flow chart (6). Bold value indicates number of participants.

It was a randomized, controlled, four-arm, parallel exercise and/or dietary intervention study (ClinicalTrials.gov: NCT03934476), with the primary aim of examining the VLCHF and HIIT effect on body composition and cardiorespiratory fitness level (6). The method for random assignment is presented in Supplemental Material. There were 91 participants allocated to the four study groups and these completed a 12-week experimental period (Figure 1). Participants were randomly allocated to four study groups: 1) high-intensity interval training (HIIT) and habitual diet, 2) very low-carbohydrate, high-fat diet (VLCHF) and habitual physical activity (no regular exercise training), 3) VLCHF diet and HIIT, and 4) Control (habitual diet and physical activity, no regular exercise training). Dual-energy X-ray absorptiometry (DXA) and graded exercise test to volitional exhaustion were used for the body composition and cardiorespiratory fitness (CRF) assessments, respectively.

To obtain measures of no intervention, a control group was utilized. Participants in the control group were advised not to change their habitual diet and physical activity regime. Therefore, no diet advice was provided.

Results for the primary outcome were previously reported, that a VLCHF diet, either in isolation or in combination with HIIT, caused a significant reduction in visceral adipose tissue (VAT) mass and body composition variables. HIIT alone did not induce such effects on body composition, but improved exercise capacity (6). We utilized the infrastructure of this trial to conduct a preplanned ancillary study focused on clinically relevant risk factors of cardiometabolic health. We analyzed blood samples following a 3-h fast before the experimental period (T_0_) and after 4, and 12 weeks (T_1_ and T_3_). Blood pressure was analyzed also after 8 weeks (T_2_). The participant set used for the primary analysis was identical with the participant set presented in this study.

### Participants

We enrolled adults aged 20–59 years with BMI 25.00–40.00 kg/m^2^, who were not engaged in any regular exercise. Participants with known chronic diseases were excluded. Additional eligibility criteria are listed in Supplementary Table 1. The participants had no previous experience with the VLCHF diet or HIIT. The recruitment details and dropouts during the study are shown in Figure 1. Written informed consent was obtained from all study participants. The study design was approved by the Ostrava university Ethics Committee (nr. 1/2018).

### High-Intensity Interval Training (HIIT)

Prior to the intervention, the participants were provided with detailed instructions on the HIIT program. This was for both the HIIT and VLCHF+HIIT groups and done both in verbally and written form. The participants were instructed to complete 3 HIIT sessions per week where one HIIT session was completed during weeks 4, 8, and 12 when the participants visited the laboratory and two were home-based and self-performed. Each HIIT session had a warm up and cool down period of 5-min of slow walking. HIIT consisted of a 3 min interval of high-intensity walking (Borg's scale RPE 18–19) followed by a 3 min interval of low-intensity walking (RPE 9–11). Participants performed 4, 6, and 8 high-intensity intervals in the first, second, and third 4-week period, respectively. Therefore, duration at high-intensity was 12, 18 and 24 min and total session time increased from 31 to 43 min and 55 min during each 4-weeks, respectively. Training intensity was measured with a heart rate monitor (Polar M430; Polar Electro, Oy, Finland). These data were subsequently uploaded to Polar Flow (Polar Electro, Finland) and analyzed regularly to track compliance. Participants were instructed to record all additional training sessions of any type in addition to the study protocol.

### Dietary Intervention

Both the HIIT and control groups were asked to maintain their habitual dietary intake without restriction. The VLCHF diet was defined as allowing no more than 50 g of carbohydrates (CHO) per day (20). Neither diet included a specific calorie or energy goal. However, participants in the VLCHF group were advised to compensate for the total energy decrease caused by CHO intake restriction by increasing their natural non-trans-fat intake (e.g., cream, butter, olive, and coconut oil). A target protein intake of 1.5 g/kg lean body mass was recommended. Contrary to the strict CHO restriction, participants were asked to keep to targets. The use of all sweetened and grain-based products had to be minimized. The recommended food included whole food sources, such as meats, vegetables, non-sweetened products, full-fat dairy items, nuts, and seeds. A dietitian provided detailed dietary advice before and during the study (on request or at least once a month). In addition, a handbook was provided to participants containing food lists, guidelines for estimating macronutrient amounts, and sample recipes. To record all foods and quantities consumed an app was used in all study groups (www.kaloricketabulky.cz). This commenced seven days before the start of the intervention. Alcoholic beverages were restricted during the intervention period, and dietary supplements were not permitted 1 month before and during the intervention period. Caffeinated beverages were restricted only before the laboratory sessions.

### Anthropometric Analysis

The results of the anthropometric analysis have previously been reported (6). In summary, the total body mass and visceral adipose tissue (VAT) mass significantly decreased in the VLCHF (by median [IQR]: −6.9 ([−8.4; −5.6]) % and −23.2 [−26.5; −14.7] %, respectively) and VLCHF+HIIT (by −9.0 [–10.9; −7.9] % and −17.6 [−23.8; −10.8] %, respectively) groups after 12 weeks despite no significant changes in the HIIT and Control groups.

### Laboratory Methods

Fasting blood samples were collected from the antecubital vein. Whole blood samples with EDTA as an anticoagulant were used immediately for blood count and HbA1c determination. Serum collection tubes were allowed to clot for 30 min and subsequently centrifuged at 2 500 g for 10 min to separate the serum. Blood serum was divided into three 1-ml aliquots, which were frozen at −80°C until analysis. The S-Monovette® system (Sarstedt, Nümbrecht, Germany) was used for blood sample collection.

Blood count parameters were measured using a UniCel® DxH™ 800 hematology analyzer (Beckman Coulter, Inc., Brea, CA, USA). Glycated hemoglobin (HbA1c) was measured using a D-10™ Bio-Rad device (Bio-Rad Laboratories, Inc., Hercules, CA, USA). Glucose, triglyceride (TG), total cholesterol, and high- and low-density lipoprotein cholesterol (HDL-C and LDL-C, respectively) concentrations were measured using an AU 5820 device (Beckman Coulter, Inc., Brea, CA, USA). Serum levels of leptin, adiponectin, TNF-α, IL−1RA, IL−1β, and IL-10 were determined by multiplex technology using a Bio-Plex MAGPIX system (Bio-Rad Laboratories, Redmond, WA, USA). Hs-IL-6 concentrations were measured using a Human IL-6 Quantikine ELISA kit (R&D Systems, Minneapolis, MN, USA) on a DSX device (Dynex Technologies, Chantilly, VA, USA). Insulin concentration was measured using a UniCel DxI 800 analyzer (Beckman Coulter, Inc., Brea, CA, USA). Lipoprotein(a) [Lp(a)] concentration was measured using a BN ProSpec system (BN ProSpec, Siemens Healthcare Diagnostics Product GmbH, Germany).

The intra-assay coefficients of variation for biochemical and blood count parameters were < 5%. Leptin, adiponectin, TNF-α, IL−1RA, IL−1β, IL−10, and hs-IL−6 were determined with an inter-assay coefficient lower than 10%.

Triglyceride-glucose (TyG) index was calculated by applying the following equation (21):


(1) $$\begin{array}{r} \text{TyGindex}=ln\left[\frac{fasting\;serum\;TG\;\times fasting\;plasma\;glucose}{2}\;\right] \end{array}$$

Homeostatic model assessment of insulin resistance (HOMA-IR) was calculated using the formula (22):


(2) $$\begin{array}{r} \text{HOMA}-\text{IR}=\frac{plasma\;glucose\;\times \;serum\;insulin}{22.5} \end{array}$$

A capillary blood sample was drawn from a finger to measure β-hydroxybutyrate (βHB) (FreeStyle Optium Neo, Oxon, United Kingdom). All participants self-analyzed it twice a week (every Monday and Thursday) in a fasting state in the morning to monitor responses to the VLCHF diet and to control for adherence.

### Blood Pressure

Systolic and diastolic blood pressure (BP) was automatically measured three times with 1–2 min apart after the participant had been sitting for ≥10 min in a quiet room by applying a standard device (Nissei DM 3000, Nihon Seimitsu Sokki Co., Japan). This procedure is in line with the recommendations of the American College of Cardiology, American Heart Association, and European Society of Hypertension (23, 24). Participants were instructed to avoid caffeinated beverages for at least 60 min before the blood pressure measurements.

### Statistical Analyses

The categorical variable (sex) was described by frequency ratio, and numerical variables were described by median and interquartile range (IQR) at each time point. Subsequently, the absolute changes of the monitored variables at time T_1_, T_2_, and T_3_ with respect to the baseline (T_0_) were analyzed. The absolute changes were tested for normal distribution using the Shapiro-Wilk test. In some cases, significant deviations from normality were detected such that non-parametric methods of data description (median and interquartile range) and statistical inference were used. Significance of change was tested by 95% confidence interval (CI) of median and two-tailed Wilcoxon signed-rank test for each variable, each group, and each time. The effect size (ES) of the observed changes was specified by the Wilcoxon effect size (*r*), including its 95% confidence interval. Threshold values for ES were 0.10 to < 0.30 (small), 0.30 to < 0.50 (medium), ≥ 0.50 (large).

Finally, the absolute changes in the given variables for the HIIT, VLCHF, HIIT+VLCHF, and Control groups were compared using the Kruskal-Wallis test at each time point. Dunn's test was used to analyze specific sample pairs for stochastic dominance. Dunn's test multiple comparison *p*-values was adjusted with the Benjamini-Hochberg method. The effect size of the observed differences was assessed using the eta squared based on the H-statistic, including its 95% confidence interval. Threshold values for ES eta squared were 0.01 to < 0.08 (small), 0.08 to < 0.26 (medium), ≥ 0.26 (large) (25).

An a priori power analysis using GPOWER (26) with power set at 0.80 and significance level set at 0.05 was calculated retrospectively. The power analysis indicated that a total sample of 76 people would be needed to detect large effects (*f* = 0.40) for this study with 4 groups. A total sample of 180 people would be needed to detect medium effects (*f* = 0.25) (27). Thus, the sample size was sufficient to reveal that a large effect could not be interpreted as non-significant.

In all cases, statistical significance was set at *p* < 0.05. Statistical analyses were performed using R Core Team (28).

## Results

### Participants

The flow chart of participants through the trial, as well as the reasons of dropouts, are depicted in Figure 1. Participant characteristics at baseline are listed in Table 1.

**Table 1:** Baseline characteristics of study participants (all Caucasian).

	**HIIT (*N* = 22)**	**VLCHF (*N* = 25)**	**VLCHF+HIIT (*N* = 25)**	**Control (*N* = 19)**	**Between-group differences**
	***M*** **(IQR)**	***M*** **(IQR)**	***M*** **(IQR)**	***M*** **(IQR)**	**p-value**
Male:Female	6:16	8:17	7:18	6:13	0.648
Age (year)	46 (38.8; 53.3)	43 (35.0; 51.5)	43 (32, 51)	40 (31; 53)	0.722
Height (cm)	167.7 (160.6; 175.0)	170.1 (164.7; 177.9)	169.8 (160.9; 179.7)	171.9 (162.8; 177.6)	0.336
BMI	28.7 (26.9; 30.9)	31.3 (27.7; 33.0)	31.0 (27.2; 35.1)	28.7 (26.7; 32.9)	0.690
WHtR	0.60 (0.54; 0.62)	0.62 (0.56; 0.65)	0.62 (0.54; 0.68)	0.60 (0.54; 0.63)	0.447
Systolic BP (mmHg)	127 (114; 137)	132 (119; 144)	128 (121; 138)	124 (121; 134)	0.105
Diastolic BP (mmHg)	77 (72; 87)	88 (77; 93)	85 (80; 91)	82 (73; 88)	0.919
Hemoglobin (g/l)	137 (135; 145)	140 (132; 150)	140 (130; 148)	137 (130; 150)	0.934
Hematocrit (%)	0.406 (0.396; 0.421)	0.411 (0.389; 0.441)	0.41 (0.378; 0.433)	0.405 (0.381; 0.435)	0.969
Erythrocytes (10^12^/l)	4.57 (4.4; 4.81)	4.72 (4.29; 5.07)	4.62 (4.36; 4.91)	4.56 (4.44; 4.88)	0.789
Thrombocytes (10^9^/l)	244 (217; 294)	241 (210; 274)	234 (200; 267)	239 (202; 280)	0.623
Leukocytes (10^9^/l)	6.5 (5.9; 7.6)	6.5 (5.9; 7.9)	6.4 (5.3; 7)	6.7 (5.4; 7.6)	0.466
HbA1c (mmol/mol)	34 (31.5; 38.5)	34 (32; 37.8)	36 (34; 38)	34 (30; 37)	0.300
Glucose (mmol/l)	5.17 (4.94; 5.4)	5.21 (5.01; 5.59)	5.12 (4.42; 5.34)	4.91 (4.65; 5.43)	0.578
Triglycerides (mmol/l)	1.18 (0.86; 1.81)	1.28 (0.86; 2.23)	0.96 (0.77; 2.18)	1.68 (0.79; 2.32)	0.410
Cholesterol (mmol/l)	5.21 (4.51; 5.93)	5.55 (5.15; 6.14)	5.11 (4.39; 6.05)	5.47 (4.91; 6.16)	0.764
HDL-C (mmol/l)	1.36 (1.17; 1.55)	1.29 (1.04; 1.53)	1.18 (1.07; 1.47)	1.32 (1.02; 1.57)	0.210
LDL-C (mmol/l)	3.3 (2.61; 3.76)	3.49 (3.03; 3.9)	3.16 (2.49; 3.59)	3.47 (2.95; 3.79)	0.386
Insulin (mU/l)	7.7 (5.2; 10.2)	9.6 (6.2; 14.6)	7.3 (5.3; 12.2)	8.2 (7.3; 14.1)	0.436
Leptin (ng/l)	5.7 (2.8; 7.4)	6.9 (3.4; 12.9)	6.2 (4.6; 12.6)	6.2 (3; 12.3)	0.579
Adiponectin (mg/l)	27 (21; 39)	41 (22; 59)	42 (16; 58)	27 (15; 59)	0.735
TG/HDL-C (–)	0.77 (0.56; 1.37)	1.07 (0.6; 2.05)	0.74 (0.55; 1.9)	1.15 (0.47; 2.32)	0.585
TyG index (–)	3.06 (2.25; 4.67)	3.24 (2.32; 5.9)	2.63 (1.86; 5.68)	4 (2.15; 5.4)	0.398
HOMA-IR (–)	1.78 (1.13; 2.5)	2.03 (1.44; 3.66)	1.51 (1.13; 3.11)	1.92 (1.51; 3.51)	0.726
Adpn/Lep (–)	5.66 (3.39; 9.98)	5.06 (3.29; 9.53)	4.19 (2.53; 9.61)	7.16 (2.24; 22.11)	0.648

*Legend: BMI, body mass index; WHtR, waist-to-height ratio; BP, blood pressure; HbA1c, glycated haemoglobin; TG, triglycerides; HDL-C/LDL-C, high/low density lipoprotein; TyG index, triglyceride-glucose index; HOMA-IR, homeostatic model assessment of insulin resistance; Adpn/Lep, adiponectin-leptin index. Data are the median (M) with interquartile range (IQR). Kruskal-Wallis test for the between-group differences*.

### Diet

Total energy intake decreased (*p* < 0.05) in the HIIT (median [95% CI]: −6.1 [−0.2; −13.4] %), VLCHF (−19.7 [−12.5; −25.2] %), and VLCHF+HIIT (−25.8 [−20.5; −28.0] %) groups. Carbohydrate intake decreased (*p* < 0.05) by −81.8 [−79.1; −82.9] % and −82.8 [−80.4; −85.7] % in the VLCHF and VLCHF+HIIT groups, respectively. Fat intake increased by 44.6 [36.1; 61.7] % and 34.8 [24.6; 47.3] % in the VLCHF and VLCHF+HIIT groups, respectively. Protein intake did not significantly change in any of the study groups. Total energy, protein, and carbohydrate intake did not significantly change in the control group, whereas fat intake decreased (*p* = 0.023, −6.0 [−1.0; −17.5] %) (Supplementary Table 2).

### High-Intensity Interval Training

There were substantial between-group differences in the training characteristics. Total training time in the HIIT and VLCHF+HIIT groups (median 1424 and 1452 min, respectively) was substantially higher than those without the HIIT intervention (VLCHF−124 min, Control−105 min). A detailed training session analysis has already been published in the parent study (6).

### Biochemical Analysis

There were no significant between-group differences in all the biochemical variables at baseline.

**Figure 2:**
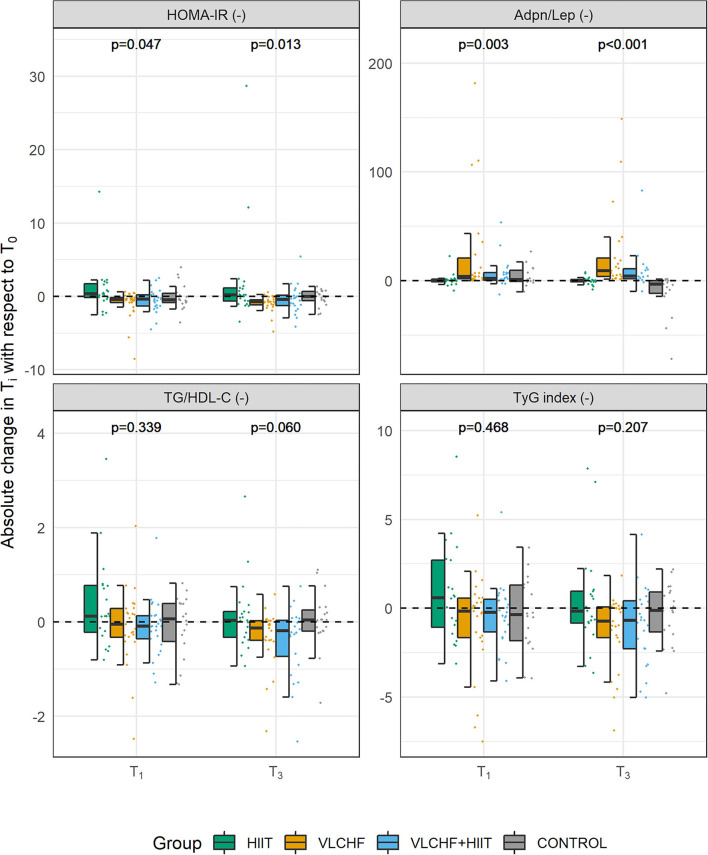
Absolute changes in HOMA-IR, Adpn/Lep ratio, TG/HDL-C ratio, and TyG index after 4 (T_1_) and 12 (T_3_) weeks.

Absolute changes in HOMA-IR after 12 weeks differed by study group (*p* = 0.013; ES 95% CI: small to large). However, no intervention group significantly differed from the Control group. The between-group significant differences were caused by the differences between HIIT and VLCHF groups. The most substantial HOMA-IR decrease was in the VLCHF group (median [IQR]: −35.7 [– 20.2; −44.0] %). This decrease in the VLCHF group was caused by changes of both HOMA-IR components insulin and glucose. Unlike glucose changes, absolute insulin changes differed by study group (*p* = 0.023, ES 95% CI: small to large) (Table 2 and Figure 2).

**Table 2:** Biochemical variables differences after 12 weeks.

	**HIIT**	**VLCHF**	**VLCHF+HIIT**	**Control**	**Between–group diff. (*p*–value)**
	**Δ*M* (95% CI)**	**Δ*M* (95% CI)**	**Δ*M* (95% CI)**	**Δ*M* (95% CI)**	
Hemoglobin (g/l)	−1.5 (−4.5; 1)	−1 (−3.5; 0)*	−4 (−6; −1.5)*	−2 (−4; 1)	0.419
Hematocrit (%)	−0.01 (−0.01; 0.00)*	0.00 (−0.01; 0.00)	−0.01 (−0.01; 0.00)*	−0.01 (−0.01; 0.00)	0.705
Erythrocytes (10^12^/l)	−0.08 (−0.16; 0.02)	−0.03 (−0.11; 0.02)	−0.12 (−0.20; −0.06)*	−0.07 (−0.17; 0.02)	0.343
Thrombocytes (10^9^/l)	2.5 (−9.5; 11.5)	−8.0 (−15.0; 6.5)	−25.0 (−29.0; −8.0)*	3.0 (−13.5; 15.0)	0.030^a^
Leukocytes (10^9^/l)	−0.40 (−0.65; 0.25)	0.00 (−0.65; 0.40)	−0.30 (−1.10; 0.15)	−0.10 (−0.85; 0.55)	0.760
HbA1c (mmol/mol)	0.0 (−2.0; 2.0)	−2.0 (−3.0; 0.0)	−2.0 (−4.0; 0.0)*	1.0 (−0.5; 3.0)	0.081
Glucose (mmol/l)	−0.02 (−0.38; 0.27)	−0.31 (−0.54; −0.05)*	0.01 (−0.50; 0.33)	−0.21 (−0.58; −0.04)*	0.316
Triglycerides (mmol/l)	0.03 (−0.33; 0.32)	−0.28 (−0.58; −0.10)*	−0.20 (−0.70; −0.01)*	0.10 (−0.41; 0.30)	0.161
Cholesterol (mmol/l)	−0.46 (−0.56; −0.05)*	−0.01 (−0.28; 0.56)	0.29 (−0.22; 0.54)	−0.21 (−0.82; 0.01)	0.063
HDL–C (mmol/l)	−0.09 (−0.19; 0.03)	0.03 (−0.08; 0.07)	0.07 (−0.05; 0.20)	−0.03 (−0.20; 0.04)	0.088
LDL–C (mmol/l)	−0.33 (−0.42; −0.08)*	0.06 (−0.12; 0.50)	0.25 (−0.04; 0.50)	−0.33 (−0.57; −0.06)*	0.003^b^
Insulin (mU/l)	0.86 (−1.61; 5.27)	−2.84 (−3.91; −1.69)**	−2.14 (−4.54; 0.37)	0.68 (−2.40; 2.32)	0.023^c^
Leptin (ng/l)	−0.19 (−1.36; 0.71)	−3.31 (−5.69; −2.59)**	−2.40 (−4.42; −1.31)*	4.07 (2.86; 5.53)**	<0.001^d^
Adiponectin (mg/l)	−1.70 (−8.48; 2.96)	9.30 (1.88; 27.85)*	3.55 (−3.85; 6.41)	3.62 (−43.18; 13.94)	0.054
TG/HDL–C (–)	0.03 (−0.21; 0.29)	−0.13 (−0.40; −0.04)*	−0.18 (−0.65; −0.03)*	0.04 (−0.16; 0.31)	0.060
TyG index (–)	−0.16 (−0.87; 10)	−0.74 (−2.11; −0.27)*	−0.68 (−1.89; 0.00)*	−0.12 (−1.18; 0.56)	0.207
HOMA–IR (–)	0.19 (−0.44; 1.24)	−0.75 (−1.13; −0.55)**	−0.44 (−1.14; 0.12)	−0.01 (−0.73; 0.38)	0.013^e^
Adpn/Lep (–)	−0.08 (−1.46; 1.03)	9.34 (6.33; 37.39)**	4.26 (2.24; 13.16)*	−3.24 (−21.27; −0.83)*	<0.001^b^

*Legend: HbA1c, glycated hemoglobin; TG, triglycerides; HDL–C/LDL–C, high/low density lipoprotein; TyG index, triglyceride–glucose index; HOMA–IR, homeostatic model assessment of insulin resistance; Adpn/Lep, adiponectin/leptin ratio*.

*Data are the median differences (ΔM) between baseline minus 12–week measures with 95% confidence intervals (CI). The complete dataset is reported in the Supplementary Material*.

Two–tailed Wilcoxon signed–rank test: * significant differences (p < 0.05) for baseline vs. 12–week; ** significant differences (p < 0.001) for baseline vs. 12–week.

*Kruskal–Wallis test for the between–group differences. Post–hoc analysis (homogenous subgroups): ^a^ – (VLCHF and VLCHF+HIIT), (HIIT, VLCHF and Control); ^b^ – (HIIT and Control), (VLCHF and VLCHF+HIIT); ^c^ – (VLCHF and VLCHF+HIIT), (HIIT, VLCHF+HIIT and Control); ^d^ – Control, HIIT, (VLCHF and VLCHF+HIIT); ^e^ – (VLCHF, VLCHF+HIIT and Control), (HIIT, VLCHF+HIIT and Control)*.

Absolute changes in the adiponectin/leptin (Adpn/Lep) ratio after 12 weeks differed by study group (p < 0.001; ES 95% CI: large) with the differences between the HIIT and Control groups vs. the VLCHF and VLCHF+HIIT groups. The Adpn/Lep ratio increased in the VLCHF group by 120.4 [88.7; 287.1] % and VLCHF+HIIT group by 158.9 [49.5; 540.2] %. These Adpn/Lep increases in the VLCHF and VLCHF+HIIT groups were caused by both the leptin decreases (*p* < 0.001; ES 95% CI: large) and adiponectin increases (*p* = 0.054; ES 95% CI: small to large) (Table 2 and Figure 2).

The TyG index and TG/HDL-C ratio significantly decreased in the VLCHF (-0.74 [-2.11; −0.27] and −0.13 [−0.40; −0.04], respectively) and VLCHF+HIIT (−0.68 [−1.89; 0.00] and −0.18 [−0.65; −0.03]) group (Table 2 and Figure 2).

Absolute changes in LDL-C after 12 weeks differed by study group (*p* = 0.003; ES 95% CI: small to large) A *post hoc* analysis revealed differences between the both diet groups (VLCHF, VLCHF+HIIT) and HIIT and Control groups. LDL-C non-significantly changed in the VLCHF group by 1.6 [−7.8; 20.0] % and in the VLCHF+HIIT group by 7.4 [−6.1; 20.3] %). However, LDL-C decreased in the HIIT (*p* = 0.012; −8.7 [−12.7; 0.3] %) and Control (*p* = 0.016; −10.1 [−18.3; −1.0] %) groups (Table 2). The complete dataset is reported in the Supplementary Material (Supplementary Tables 3–5).

Lp(a), TNF-α, hs-IL−6, IL−1RA, IL−1β, and IL-10 remained mostly under a detection level of the assay. Therefore, no further statistical analyses were conducted (Supplementary Table 6).

There were substantial increases in β-hydroxybutyrate concentration (βHB) in the VLCHF and VLCHF+HIIT groups. The highest βHB concentrations were achieved after 2 weeks of VLCHF diet intervention. βHB concentrations in the HIIT and control groups remained within the range between 0.0 to 0.3 mmol/l for the whole 12-week intervention. A detailed βHB analysis has already been presented in the parent study (6).

### Blood Pressure

Systolic BP decreased (p < 0.05) in all three intervention groups after 8 weeks, when compared to the baseline level, and remained significantly decreased after 12 weeks in the VLCHF+HIIT group. However, systolic BP did not significantly differ by study group at any time point. The between-group differences were shown in diastolic BP after 12 weeks (*p* = 0.049, ES 95% CI: small to medium), with the most pronounced decreases in the VLCHF (Δ*M* [95% CI]: −4.0 [−6.8; −0.3] mmHg; ES 95% CI: small to large) and VLCHF+HIIT (−5.3 [−8.0 −3.3] mmHg; ES 95% CI: large) groups (Table 3).

**Table 3:** Blood pressure outcomes.

	**HIIT**	**VLCHF**	**VLCHF+HIIT**	**Control**	**Between–group diff. (*p*–value)**
**Systolic**					
PRE	127 (114; 137)	132 (119; 144)	128 (121; 138)	124 (121; 134)	–
4 weeks	121 (112; 134)	126 (118; 132)*	125 (115; 134)	124 (117; 131)	0.339
8 weeks	118 (112; 134)*	124 (114; 133)*	120 (114; 134)*	120 (112; 126)	0.990
12 weeks	124 (115; 140)	127 (117; 138)	125 (115; 130)*	120 (118; 133)	0.236
**Diastolic**					
PRE	77 (72; 87)	88 (77; 93)	85 (80; 91)	82 (73; 88)	–
4 weeks	79 (72; 87)	82 (79; 87)	81 (77; 90)*	79 (74; 86)	0.137
8 weeks	75 (69; 84)*	83 (79; 88)	80 (72; 86)**	77 (71; 83)	0.380
12 weeks	80 (72; 87)	83 (75; 89)*	81 (73; 86)**	79 (73; 82)	0.049^a^

Legend, * different from the baseline (PRE) at p < 0.05.

*Values are shown as median (interquartile range)*.

Two–tailed Wilcoxon signed–rank test, * significant differences (p < 0.05) to baseline (PRE); ** significant differences (p < 0.001) to baseline (PRE).

*Kruskal–Wallis test for the between–group differences. Post–hoc analysis (homogenous subgroups): ^a^ – (HIIT,VLCHF and Control), (VLCHF, VLCHF+HIIT and Control)*.

## Discussion

In this randomized controlled trial, we found that VLCHF diet, when compared to HIIT, over 12 weeks in individuals with overfat constitution had substantial benefits for chronic non-communicable diseases risk factors HOMA-IR, Adpn/Lep ratio and diastolic BP beside the already presented decrease in body mass and VAT (6). However, we did not find significant changes of HOMA-IR from the Control group. Adding HIIT to the VLCHF diet did not caused an extra effect on these variables. We showed that VLCHF diet improved insulin sensitivity and skewed the leptin and adiponectin levels toward anti-inflammatory phenotypes. Despite significant increases in saturated fat intake, we found no significant elevation of LDL-C in the VLCHF and VLCHF+HIIT groups.

### VLCHF Diet and βHB

Less than 50 g/day of CHO was required for the VLCHF groups in this study (29). The aim of the VLCHF diet intervention was not to reduce total energy intake. As such the VLCHF and VLCHF+HIIT groups were encouraged to compensate for the CHO intake restriction by increasing fat intake while maintaining protein consumption. Nevertheless, fat intake was insufficient to keep the total energy intake unchanged (Supplementary Table S2). This is indeed a common situation in real-life conditions. ßHB measures confirmed adherence to the diet in both VLCHF and VLCHF+HIIT groups as has been shown already (13). Notably, total energy intake significantly decreased in the HIIT group despite no diet modification. This effect was likely due to an increased interest in a healthy lifestyle when participating in such a research study.

### HOMA-IR

We found that the HOMA-IR, a frequently used index to evaluate insulin resistance, was substantially reduced after 12 weeks of the VLCHF diet, when compared to HIIT. We showed this improved insulin sensitivity even if the participants with overfat constitution were without diabetes and within the normal range of HbA1c. Low carbohydrate diets proved to be more effective than higher carbohydrate (low fat) diets in improving fasting glucose and insulin and insulin sensitivity as measured by HOMA-IR in individuals with obesity and insulin resistance (9) and patients with obesity and non-alcoholic fatty liver disease (10). Not surprisingly, low carbohydrate diets are associated with a large (32 %) increase in remission of diabetes (7). Insulin resistance and excessive body fat are considered among the most important causes of several chronic metabolic and cardiovascular diseases. The cellular and physiological mechanisms are complex and involve adiposity-induced alterations in β cell function, adipose tissue biology, and multi-organ insulin resistance. All these perspectives can be improved with adequate body mass loss (30), which we also showed in this study (6) which may have contributed to the HOMA-IR reduction.

Another surrogate measure for the diagnosis of insulin resistance is the TyG index, which is independently and more strongly associated with arterial stiffness in patients with type 2 diabetes than HOMA-IR (31). The TyG index significantly decreased in both the VLCHF group by median 25.9 [IQR: −38.0; 1.2] % and VLCHF+HIIT group by median 23.1 [−54.7; 27.0] %, when the 12-week outcomes were compared to the baseline. However, these favorable changes were not sufficient to prove a significant between-group differences (*p* = 0.207). Nevertheless, we can suggest that a carbohydrate intake restriction might be beneficial not only for the visceral adipose tissue reduction as we showed in the parent study (6), but also for the treatment of the impaired insulin resistance, as already shown elsewhere (29, 32, 33).

The uniqueness of this study, however, lay in including HIIT, alone or in combination with VLCHF diet, into consideration. An exercise intervention program proved to be effective in the treatment of insulin resistance in individuals with overweight/obesity (34), metabolic syndrome (35) or type 2 diabetes, i.e., reduces fasting insulin, HOMA-IR, fasting blood sugar, HbA1c, and body mass index (36). However, we did not show any or additional effect of HIIT on these variables after 12 weeks. This inconsistency can be related to participants characteristics, study duration or other design issues. A diet adjustment seems to be, therefore, crucial within any lifestyle modification for body mass management and reducing health risk variables, despite we still consider physical activity and regular exercise important, e.g., for the maintenance or improvement of the CRF level (6). There is a solid evidence that the CRF level is inversely associated with all-cause, CVD and cancer mortality. A dose-response analyses even showed that a per one-MET increase of the CRF level was associated with 12 %, 13 %, and 7 % reduced risk of all-cause, CVD and cancer mortality (37).

### Adpn/Lep Ratio

We showed significant beneficial changes in the leptin and adiponectin concentrations in the VLCHF group after 12 weeks. The significant decrease in leptin levels also occurred in the VLCHF+HIIT group, whereas the adiponectin increase was not significant. No such beneficial changes were detected in the HIIT and Control groups. A low adiponectin-leptin ratio has been proposed as a promising marker of adipose tissue dysfunction and may lead to chronic systemic inflammation. Leptin is involved in inflammatory responses, and its increased levels are induced by adiposity. In contrast, a decrease in adiposity leads to increased adiponectin levels, which is considered an anti-inflammatory marker (38, 39).

We have already demonstrated favorable changes in serum adiponectin and leptin concentrations in healthy young individuals following a comparable 12-week VLCHF diet (16). The present study shows that a VLCHF diet can induce similar changes in individuals with overfat constitution. Unlike our previous study with healthy young individuals, in which body mass reduction was only small (16), the decrease in leptin and increase in adiponectin levels in this study might be associated with the large body mass changes in both VLCHF and VLCHF+HIIT groups, which were not present in the HIIT and Control groups. Since the diet intervention in the present study induced a substantial increase in βHB (6), the beneficial changes in leptin and adiponectin could also be related to the anti-inflammatory effect of βHB. βHB inhibits histone deacetylase enzymes, free fatty acid receptors, and the NLRP3 inflammasome, inhibiting inflammation, oxidative stress, and the development of chronic diseases (40, 41).

We also assessed other biomarkers associated with low-grade chronic inflammation, since an excess dietary intake of both CHO and lipids is considered harmful (42, 43). Neither anti-inflammatory (IL-1RA, IL-10) nor pro-inflammatory (TNF-α, IL-6, IL−1β) cytokines were analyzed because they mostly remained under the clinically relevant levels at baseline and in response to all interventions. Therefore, from these specific perspectives, a 12-week CHO restricted high-fat diet cannot be considered harmful.

### LDL-C

A common concern about the VLCHF diet is its potential atherogenicity due to the high dietary fat intake and subsequent elevated LDL-C levels (44, 45). An elevated LDL-C is traditionally considered a risk factor for atherosclerotic cardiovascular disease (ASCVD) (46). However, a harmful effect of the LDL-C, as well as an elevated LDL-C level induced by the increased saturated fat intake (a.k.a. diet heart hypothesis), have been challenged (47, 48). Paradoxically, it has been shown that those individuals with the high levels of LDL-C live just long or longer than those with low LDL-C (49, 50). A low LDL-C level can even be interpreted as a biomarker of illness severity (51). Therefore, not surprisingly, the LDL-C level is not directly considered among the most important factors for the latest QRISK®3 calculator of a cardiovascular disease risk estimation (52). Unlike our previous study in healthy young individuals (16), we did not show significant LDL-C changes in both the VLCHF and VLCHF+HIIT groups in the individuals with overfat composition despite a large increase in SFA intake (Supplementary Table 2). The significant between-group differences were caused by LDL-C decrease in the HIIT and Control groups. However, substantial inter-individual differences in LDL-C levels were observed and needs to be considered.

### TG/HDL-C Ratio

The rationale for population-wide restriction of dietary SFA to prevent cardiovascular diseases and reduced mortality is not supported by the results of randomized clinical trials and observational cohort studies (53). SFAs appear to be less harmful than the proposed polyunsaturated alternatives because of their resistance to lipid peroxidation associated with oxidative stress and inflammation (54). In addition, a standard lipid panel analysis may not be sufficient to consider cardiovascular risk (55). An assessment of the LDL-C and HDL-C particle size attracted a research attention because the cardiovascular risk was shown to be associated only with the small, dense LDL-C and HDL-C particle sizes (53, 56). Unfortunately, this analysis is highly demanding and was not performed in this study. However, the triglyceride/HDL-C ratio can predict particle size and is closely related to cardiometabolic diseases (57). In the current study, triglyceride levels and HDL-C tended to change in a favorable manner in the VLCHF and VLCHF+HIIT groups. The triglyceride/HDL-C ratio was therefore lower in both the VLCHF (*p* = 0.011; median [IQR]: −11.9 [−34.1; 7.2] %) and VLCHF+HIIT (*p* = 0.037; −24.3 [−37.6; 6.9] %) groups when comparing the 12-week outcomes to the baseline. However, these changes were not sufficient to cause significant between group differences (*p* = 0.06).

### Blood Pressure (BP)

We revealed significant between-group differences in diastolic BP after 12 weeks. *Post-hoc* analysis showed that diastolic BP decreased in the VLCHF and VLCHF+HIIT groups by median 4.6 (IQR: [−8.6; 2.4]) % and 7.0 [−9.8; −3.5] %, respectively. However, a recent systematic review and meta-analysis found no significant differences in BP changes between low carbohydrate high fat diets and low-fat high carbohydrate diets. The studies included in this review lasted from several weeks to 1 year (58). In contrary, significant favorable changes in both systolic and diastolic BP were shown in patients with type 2 diabetes or impaired glucose tolerance after an average of 2 years observation (IQR 10–32 months) of a low carbohydrate diet (59). Similarly to our findings, a 4-week carbohydrate restriction diet lowered systolic BP in women with obesity/overweight, although the combination with exercise (HIIT or MICT, i.e., moderate-intensity continuous training) had no additional benefits (60). The presented findings cannot, however, be unconditionally ascribed to the VLCHF diet. Again, the body mass loss, induced by the VLCHF diet in this study (as well as in all the cited studies above), could substantially contribute to the presented diastolic BP changes.

### Strengths and Limitations

The VLCHF diet and HIIT are popular diet and exercise approaches. The main strength is their independent and combined investigation within one randomized clinical trial with sufficient statistical power, high participant adherence, and focus on cardiometabolic risk factors in the overfat population. We wanted to show a complete picture of these effects caused by the VLCHF diet and HIIT. Therefore, another strength of this trial is considering several perspectives associated with chronic non-communicable diseases, as also presented in the parent study (6). Finally, the real-life design of this study shows expectable participants responses to these diets and exercise lifestyle modifications.

There are some limitations of this study. The real-life application did not allow to collect some of the data in person (e.g., daily records of nutrition and exercise sessions). As such, the participant's adherence to all study requirements cannot be fully controlled. However, compliance as well as controlling intensity and duration of the HIIT sessions were done by regular checks of the HR records. Also, despite the very detailed diet instructions, diets could differ in many ways, such as the ratio and type of fatty acids, fiber type and amount, glycemic index, food processing, micronutrient content, as well as food timing and frequency. Furthermore, the study groups were not balanced for sex and, despite the randomization, the median age of the HIIT group was slightly higher than in the other study groups. Furthermore, the menstrual cycle and menopause status were not considered within the data collection and analysis. Another limitation is that there are between individuals' differences in the daytime of the blood withdraw (early morning to afternoon) and possible circadian rhythms have not been considered. However, each participant performed the laboratory sessions (blood withdraw included) during similar day hours (± 60 min). The adiponectin analysis was based on the total adiponectin only. Other forms of adiponectin were not considered. The sample size allowed us to be confident about revealing significant large effect. However, potential significant small or medium effects could be interpreted as non-significant. The statistical significance outcomes for HOMA-IR, Adpn/Lep ratio, and LDL-C were sufficiently robust, but between-group changes in diastolic BP (*p* = 0.049) are borderline and need to be interpreted cautiously, although some prior studies are supportive (59, 60). The presented results might be related to the body composition changes, which we showed in the parent study (6). However, this analysis is beyond the scope and aim of this study. We have to highlight also the interindividual differences in all the variables, despite the group statistical analysis revealed significant between group differences and changes from the baseline (Figure 2). The “one size does not fit all” approach needs to be considered and requires further investigation. Finally, even if we have no evidence of a detrimental effect of the VLCHF diet, long-term studies and individually adjusted carbohydrate intake restriction is needed for safety and life-long maintenance in various populations. Additionally, although the HIIT approach did not show additional effects, we need to consider other types and amounts of exercise with respect to the evidence regarding positive effects of exercise on health in the literature.

## Conclusions

We extended the preliminary findings about beneficial effect of the VLCHF diet on body composition by investigating metabolic and cardiovascular risk factors. A 12-week VLCHF diet intervention in individuals with overfat constitution is effective for favorable changes in HOMA-IR (compared to HIIT), Adpn/Lep ratio, and diastolic BP. HIIT, or HIIT combined with the VLCHF diet, had no additional benefits for the analyzed variables. No adverse side effects were observed.

## Data Availability Statement

The original contributions presented in the study are included in the article/Supplementary Material, further inquiries can be directed to the corresponding author.

## Ethics Statement

The studies involving human participants were reviewed and approved by The University of Ostrava. The patients/participants provided their written informed consent to participate in this study.

## Author Contributions

LC and TD designed the study, collected, analyzed, revised, and submitted the manuscript. ML analyzed the data. DP, PM, PH, and PL designed the study. ML, DP, PM, PH, and PL revised the manuscript. LC, TD, ML, DP, PM, PH, and PL interpreted the data and drafted the manuscript. All authors approved the final version of the manuscript.

## Funding

This work was supported by the Czech Science Foundation (Grant 18-08358S). The laboratory facilities used for this research were bought with the support of the Healthy Aging in Industrial Environment project (registration number CZ.02.1.01/0.0/0.0/16_019/0000798, founded by the European Union via the Ministry of Education, Youth and Sports of the Czech Republic). Statistical analysis was supported by Grant of SGS No. SP2022/6, VŠB—Technical University of Ostrava, Czech Republic.

## Conflict of Interest

The authors declare that the research was conducted in the absence of any commercial or financial relationships that could be construed as a potential conflict of interest.

## Publisher's Note

All claims expressed in this article are solely those of the authors and do not necessarily represent those of their affiliated organizations, or those of the publisher, the editors and the reviewers. Any product that may be evaluated in this article, or claim that may be made by its manufacturer, is not guaranteed or endorsed by the publisher.

## References

[1] U.S. Department of Health and Human Services, Centers for Disease Control and Prevention NC for HS. Health, United States, 2015: With Special Feature on Racial and Ethnic Health Disparities. Hyattsville, MD: Frontiers in Public Health (2016).

[2] Maffetone PB Laursen PB. The prevalence of overfat adults and children in the US. Front Public Heal. (2017) 5:1–9. 10.3389/fpubh.2017.00290 29164096 PMC5671970

[3] Maffetone PB Rivera-Dominguez I Laursen PB. Overfat and underfat: new terms and definitions long overdue. Front Public Heal. (2017) 4:1–10. 10.3389/fpubh.2016.00279 28097119 PMC5206235

[4] Okorodudu DO Jumean MF Montori VM Romero-Corral A Somers VK Erwin PJ. Diagnostic performance of body mass index to identify obesity as defined by body adiposity: a systematic review and meta-analysis. Int J Obes. (2010) 34:791–9. 10.1038/ijo.2010.5 20125098

[5] Moriyama K. Associations between the triglyceride to high-density lipoprotein cholesterol ratio and metabolic syndrome, insulin resistance, and lifestyle habits in healthy Japanese. Metab Syndr Relat Disord. (2020) 18:260–6. 10.1089/met.2019.0123 32191558

[6] Cipryan L Dostal T Litschmannova M Hofmann P Maffetone PB Laursen PB. Effects of a very low-carbohydrate high-fat diet and high-intensity interval training on visceral fat deposition and cardiorespiratory fitness in overfat individuals: a randomized controlled clinical trial. Front Nutr. (2021) 8:1–13. 10.3389/fnut.2021.785694 34993222 PMC8724307

[7] Goldenberg JZ Day A Brinkworth GD Sato J Yamada S Jönsson T. Efficacy and safety of low and very low carbohydrate diets for type 2 diabetes remission: systematic review and meta-analysis of published and unpublished randomized trial data. BMJ. (2021) 372:m4743. 10.1136/bmj.m4743 33441384 PMC7804828

[8] Veldhorst MA Westerterp-Plantenga MS. & Westerterp KR. Gluconeogenesis and energy expenditure after a high-protein, carbohydrate-free diet. Am J Clin Nutr. (2009) 90:519–26. 10.3945/ajcn.2009.27834 19640952

[9] O'Neill BJ. Effect of low-carbohydrate diets on cardiometabolic risk, insulin resistance, and metabolic syndrome. Curr Opin Endocrinol Diabetes Obes. (2020) 27:301–7. 10.1097/MED.0000000000000569 32773574

[10] Watanabe M Risi R Camajani E Contini S Persichetti A Tuccinardi D. Baseline homa IR and circulating FGF21 levels predict NAFLD improvement in patients undergoing a low carbohydrate dietary intervention for weight loss: a prospective observational pilot study. Nutrients. (2020) 12:1–13. 10.3390/nu12072141 32708435 PMC7400878

[11] Iaccarino G Franco D Sorriento D Strisciuglio T Barbato E Morisco C. Modulation of insulin sensitivity by exercise training: implications for cardiovascular prevention. J Cardiovasc Transl Res. (2021) 14:256–70. 10.1007/s12265-020-10057-w 32737757 PMC8043859

[12] Yaribeygi H Atkin SL Simental-Mendía LE Sahebkar A. Molecular mechanisms by which aerobic exercise induces insulin sensitivity. J Cell Physiol. (2019) 234:12385–92. 10.1002/jcp.28066 30605232 PMC13484081

[13] Verheggen RJHM Konstanti P Smidt H Hermus ARMM Thijssen DHJ Hopman MTE. Eight-week exercise training in humans with obesity: Marked improvements in insulin sensitivity and modest changes in gut microbiome. Obesity. (2021) 29:1615–24. 10.1002/oby.23252 34467673 PMC9291576

[14] Heiston EM Liu Z Ballantyne A Kranz S Malin SK. A single bout of exercise improves vascular insulin sensitivity in adults with obesity. Obesity. (2021) 29:1487–96. 10.1002/oby.23229 34339111 PMC8387339

[15] Ryan BJ Schleh MW Ahn C Ludzki AC Gillen JB Varshney P. Moderate-Intensity exercise and high-intensity interval training affect insulin sensitivity similarly in obese adults. J Clin Endocrinol Metab. (2020) 105:e2941–59. 10.1210/clinem/dgaa345 32492705 PMC7347288

[16] Cipryan L Dostal T Plews DJ Hofmann P Laursen PB. Adiponectin/leptin ratio increases after a 12-week very low-carbohydrate, high-fat diet, and exercise training in healthy individuals: a non-randomized, parallel design study. Nutr Res. (2021) 87:22–30. 10.1016/j.nutres.2020.12.012 33596508

[17] Lee HS Lee J. Effects of combined exercise and low carbohydrate ketogenic diet interventions on waist circumference and triglycerides in overweight and obese individuals: a systematic review and meta-analysis. Int J Environ Res Public Health. (2021) 18:828. 10.3390/ijerph18020828 33478022 PMC7835865

[18] Hu J Wang Z Lei B Li J Wang R. Effects of a low-carbohydrate high-fat diet combined with high-intensity interval training on body composition and maximal oxygen uptake: a systematic review and meta-analysis. Int J Environ Res Public Health. (2021) 18:10740. 10.3390/ijerph182010740 34682481 PMC8535842

[19] Laguzzi F Maitusong B Strawbridge RJ Baldassarre D Veglia F Humphries SE. Intake of food rich in saturated fat in relation to subclinical atherosclerosis and potential modulating effects from single genetic variants. Sci Rep. (2021) 11:7866. 10.1038/s41598-021-86324-w 33846368 PMC8042105

[20] Feinman RD Pogozelski WK Astrup A Bernstein RK Fine EJ Westman EC. Dietary carbohydrate restriction as the first approach in diabetes management: Critical review and evidence base. Nutrition. (2015) 31:1–13. 10.1016/j.nut.2014.06.011 25287761

[21] Park HM Lee HS Lee Y-J Lee J-H. The triglyceride–glucose index is a more powerful surrogate marker for predicting the prevalence and incidence of type 2 diabetes mellitus than the homeostatic model assessment of insulin resistance. Diabetes Res Clin Pract. (2021) 180:109042. 10.1016/j.diabres.2021.109042 34506839

[22] Dimova R Chakarova N Grozeva G Tankova T. Evaluation of the relationship between cardiac autonomic function and glucose variability and HOMA-IR in prediabetes. Diabetes Vasc Dis Res. (2020) 17:147916412095861. 10.1177/1479164120958619 32985241 PMC7919217

[23] Casey DE Thomas RJ Bhalla V Commodore-Mensah Y Heidenreich PA Kolte D. 2019 AHA/ACC clinical performance and quality measures for adults with high blood pressure: a report of the American College of Cardiology/American Heart Association Task Force on performance measures. Circ Cardiovasc Qual Outcomes. (2019) 12:e000057. 10.1161/HCQ.0000000000000057 31714813 PMC7717926

[24] Parati G Stergiou GS Asmar R Bilo G de Leeuw P Imai Y. European Society of Hypertension guidelines for blood pressure monitoring at home: a summary report of the second international consensus conference on home blood pressure monitoring. J Hypertens. (2008) 26:1505–26. 10.1097/HJH.0b013e328308da66 18622223

[25] Tomczak M Tomczak E. The need to report effect size estimates revisited. an overview of some recommended measures of effect size. Trends Sport Sci. (2014) 1:19–25.

[26] Faul F Erdfelder E Lang A-G Buchner A. G^*^Power 3: a flexible statistical power analysis program for the social, behavioral, and biomedical sciences. Behav Res Methods. (2007) 39:175–91. 10.3758/BF03193146 17695343

[27] Cohen J. Statistical Power Analysis for the Behavioural Sciences. 2nd edition. Hillsdale, NJ: Erlbau (1988).

[28] R Core Team. (2020) Available at: https://www.r-project.org/

[29] Lennerz BS Koutnik AP Azova S Wolfsdorf JI Ludwig DS. Carbohydrate restriction for diabetes: rediscovering centuries-old wisdom. J Clin Invest. (2021) 131:e142246. 10.1172/JCI142246 33393511 PMC7773350

[30] Klein S Gastaldelli A Yki-Järvinen H Scherer PE. Why does obesity cause diabetes? Cell Metab. (2022) 34:11–20. 10.1016/j.cmet.2021.12.012 34986330 PMC8740746

[31] Wang S Shi J Peng Y Fang Q Mu Q Gu W. Stronger association of triglyceride glucose index than the HOMA-IR with arterial stiffness in patients with type 2 diabetes: a real-world single-centre study. Cardiovasc Diabetol. (2021) 20:82. 10.1186/s12933-021-01274-x 33888131 PMC8063289

[32] Ebbeling CB Knapp A Johnson A Wong JMW Greco KF Ma C. Effects of a low-carbohydrate diet on insulin-resistant dyslipoproteinemia—a randomized controlled feeding trial. Am J Clin Nutr. (2021) 115:154–62. 10.1093/ajcn/nqab287 34582545 PMC8755039

[33] Ludwig DS Aronne LJ Astrup A de Cabo R Cantley LC Friedman MI. The carbohydrate-insulin model: a physiological perspective on the obesity pandemic. Am J Clin Nutr. (2021) 114:1873–85. 10.1093/ajcn/nqab270 34515299 PMC8634575

[34] Battista F Ermolao A Baak MA Beaulieu K Blundell JE Busetto L. Effect of exercise on cardiometabolic health of adults with overweight or obesity: Focus on blood pressure, insulin resistance, and intrahepatic fat—a systematic review and meta-analysis. Obes Rev. (2021) 22:1–15. 10.1111/obr.13269 33960110 PMC8365642

[35] Gallo-Villegas J Castro-Valencia LA Pérez L Restrepo D Guerrero O Cardona S. Efficacy of high-intensity interval- or continuous aerobic-training on insulin resistance and muscle function in adults with metabolic syndrome: a clinical trial. Eur J Appl Physiol. (2022) 122:331–44. 10.1007/s00421-021-04835-w 34687360

[36] Sampath Kumar A Maiya AG Shastry BA Vaishali K Ravishankar N Hazari A. Exercise and insulin resistance in type 2 diabetes mellitus: a systematic review and meta-analysis. Ann Phys Rehabil Med. (2019) 62:98–103. 10.1016/j.rehab.2018.11.001 30553010

[37] Han M Qie R Shi X Yang Y Lu J Hu F. Cardiorespiratory fitness and mortality from all causes, cardiovascular disease and cancer: dose–response meta-analysis of cohort studies. Br J Sports Med. (2022) 26:bjsports-2021–104876. 10.1136/bjsports-2021-104876 35022163

[38] Frühbeck G Catalán V Rodríguez A Gómez-Ambrosi J. Adiponectin-leptin ratio: a promising index to estimate adipose tissue dysfunction. relation with obesity-associated cardiometabolic risk. Adipocyte. (2018) 7:57–62. 10.1080/21623945.2017.1402151 29205099 PMC5915018

[39] Milling S. Adipokines and the control of mast cell functions: from obesity to inflammation? Immunology. (2019) 158:1–2. 10.1111/imm.13104 31429086 PMC6700462

[40] Møller N. Ketone Body, 3-hydroxybutyrate: minor metabolite - major medical manifestations. J Clin Endocrinol Metab. (2020) 29:2341–86. 10.1210/clinem/dgaa370 32525972

[41] Youm Y-H Nguyen KY Grant RW Goldberg EL Bodogai M Kim D. The ketone metabolite β-hydroxybutyrate blocks NLRP3 inflammasome-mediated inflammatory disease. Nat Med. (2015) 21:263–9. 10.1038/nm.3804 25686106 PMC4352123

[42] Ceriello A Genovese S. Atherogenicity of postprandial hyperglycemia and lipotoxicity. Rev Endocr Metab Disord. (2016) 17:111–6. 10.1007/s11154-016-9341-8 26880302

[43] Kroemer G López-Otín C Madeo F de Cabo R. Carbotoxicity-Noxious effects of carbohydrates. Cell. (2018) 175:605–14. 10.1016/j.cell.2018.07.044 30340032 PMC6265656

[44] Urbain P Strom L Morawski L Wehrle A Deibert P Bertz H. Impact of a 6-week non-energy-restricted ketogenic diet on physical fitness, body composition and biochemical parameters in healthy adults. Nutr Metab. (2017) 14:1–11. 10.1186/s12986-017-0175-5 28239404 PMC5319032

[45] Rosenbaum M Hall KD Guo J Ravussin E Mayer LS Reitman ML. Glucose and lipid homeostasis and Inflammation in humans following an isocaloric Ketogenic diet. Obesity. (2019) 27:971–98. 10.1002/oby.22468 31067015 PMC6922028

[46] Borén J Chapman MJ Krauss RM Packard CJ Bentzon JF Binder CJ. Low-density lipoproteins cause atherosclerotic cardiovascular disease: pathophysiological, genetic, and therapeutic insights: a consensus statement from the European atherosclerosis society consensus panel. Eur Heart J. (2020) 41:2313–30. 10.1093/eurheartj/ehz962 32052833 PMC7308544

[47] Ravnskov U Diamond DM Hama R Hamazaki T Hammarskjöld B Hynes N. Lack of an association or an inverse association between low-density-lipoprotein cholesterol and mortality in the elderly: a systematic review. BMJ Open. (2016) 6:e010401. 10.1136/bmjopen-2015-010401 27292972 PMC4908872

[48] Ravnskov U de Lorgeril M Diamond DM Hama R Hamazaki T Hammarskjöld B. LDL-C does not cause cardiovascular disease: a comprehensive review of the current literature. Expert Rev Clin Pharmacol. (2018) 11:959–70. 10.1080/17512433.2018.1519391 30198808

[49] Ramsden CE Zamora D Majchrzak-Hong S Faurot KR Broste SK Frantz RP. Re-evaluation of the traditional diet-heart hypothesis: analysis of recovered data from minnesota coronary experiment (1968–73). BMJ. (2016) 353:i1246. 10.1136/bmj.i1246 27071971 PMC4836695

[50] Ravnskov U de Lorgeril M Diamond D Hama R Hamazaki T Hammarskjöld B. The LDL paradox - higher LDL cholesterol is associated with greater longevity. Ann Epidemiol Public Heal. (2020) 3:1040.

[51] Budzyński J Tojek K Wustrau B Czerniak B Winiarski P Korzycka-Wilińska W. The “cholesterol paradox” among inpatients – retrospective analysis of medical documentation. Arch Med Sci - Atheroscler Dis. (2018) 3:46–57. 10.5114/amsad.2018.74736 30775589 PMC6374572

[52] Hippisley-Cox J Coupland C Brindle P. Development and validation of QRISK3 risk prediction algorithms to estimate future risk of cardiovascular disease: prospective cohort study. BMJ. (2017) 357:1–21. 10.1136/bmj.j2099 28536104 PMC5441081

[53] Astrup A Magkos F Bier DM Brenna JT de Oliveira Otto MC Hill JO. Saturated fats and health: a reassessment and proposal for food-basedrecommendations: JACC state-of -the-art review. J Am Coll Cardiol. (2020) 76:844-57. 10.1016/j.jacc.2020.05.077 32562735

[54] Lawrence GD. Perspective: the saturated fat–unsaturated oil dilemma: relations of dietary fatty acids and serum cholesterol, atherosclerosis, inflammation, cancer, and all-cause mortality. Adv Nutr. (2021) 12:647–56. 10.1093/advances/nmab013 33693484 PMC8166560

[55] Norwitz NG Loh V. A Standard lipid panel is insufficient for the care of a patient on a high-fat, low-carbohydrate ketogenic diet. Front Med. (2020) 7:00097. 10.3389/fmed.2020.00097 32351962 PMC7174731

[56] Dugani SB Moorthy MV Li C Demler O V Alsheikh-Ali AA. Association of lipid, inflammatory, and metabolic biomarkers with age at onset for incident coronary heart disease in women. JAMA Cardiol. (2021) 6:437–47: 10.1001/jamacardio.2020.7073 33471027 PMC7818181

[57] Nam K-W Kwon H-M Jeong H-Y Park J-H Kwon H Jeong S-M. High triglyceride/HDL cholesterol ratio is associated with silent brain infarcts in a healthy population. BMC Neurol. (2019) 19:147. 10.1186/s12883-019-1373-8 31266453 PMC6604433

[58] Yang Q Lang X Li W Liang Y. The effects of low-fat, high-carbohydrate diets vs. low-carbohydrate, high-fat diets on weight, blood pressure, serum liquids and blood glucose: a systematic review and meta-analysis. Eur J Clin Nutr. (2022) 76:16–27. 10.1038/s41430-021-00927-0 34168293

[59] Unwin Tobin Murray Delon Brady. Substantial and sustained improvements in blood pressure, weight and lipid profiles from a carbohydrate restricted diet: an observational study of insulin resistant patients in primary care. Int J Environ Res Public Health. (2019) 16:2680. 10.3390/ijerph16152680 31357547 PMC6695889

[60] Sun S Kong Z Shi Q Zhang H Lei O-K Nie J. Carbohydrate restriction with or without exercise training improves blood pressure and insulin sensitivity in overweight women. Healthcare. (2021) 9:637. 10.3390/healthcare9060637 34072093 PMC8229341

