# Supplementary material for: Very Low-Carbohydrate High-Fat Diet Improves Risk Markers for Cardiometabolic Health More Than Exercise in Men and Women With Overfat Constitution: Secondary Analysis of a Randomized Controlled Clinical Trial
Source: Front Nutr. 2022 May 23;9:867690. doi: 10.3389/fnut.2022.867690 (PMC9168912; doi:10.3389/fnut.2022.867690)
Supplement: Supplementary file 1 [file Table_1.docx]

**Supplementary Methods**

Random Assignment

Participants who successfully met inclusion criteria become acquainted with all study requirements. Those who agreed to follow were eligible for randomization. The randomization was stratified by age (20-29, 30-39, 40-49, 50-59 years) and sex (male, female). A set of blinded envelopes with study group number (1-4) were prepared by a principal researcher. To ensure close balance among four study groups at any point in the study. The study group assignments were randomly permuted within blocks of 8 participants (2 participants randomly allocated to each study arm). A specific ID were created for each participant and the key was kept accessible only to staff responsible for data collection.

Statistical Analysis – Rationale for Fitting the statistical approach

We have chosen the statistical analysis related to the *a priori* defined study question. The primary aim was to investigate a 12-week effect of the HIIT and VLCHF diet on these presented variables. Therefore, we focus almost solely on this 12-week (i.e. pre-post) effect within the results, discussion, and conclusions.

However, we also performed control measurements after 4 (and 8) weeks. These measurements are considered just additional and barely discussed within the text to make the main message, i.e. 12-week effect, clear and simple. The 4-week outcomes are mostly presented in the Supplementary Material. The presented differences of dependent variables in the diet groups after 12 weeks are robust and considering 4 (and 8-week) outcomes will not change the study conclusions.

Considering all these facts together, we have used a nonparametric one-way ANOVA, i.e. Kruskal-Wallis test, for analysis between-group differences in changes (pre-post) after 12 weeks.

**Table S1. Participant eligibility criteria**

| Inclusion criteria |
| --- |
| - males. females - age 20-59 years - BMI 25.00 – 40.00 kg/m^2^ - non-smokers - no excessive alcohol intake (self-reported, ≥ to 2 drinks/day for men or ≥1 drink/day for women) - willing to accept random assignment - low active individuals over the last 1 year, i.e. no specific sports training or regular exercise - no evidence of liver, renal, metabolic, and cardiopulmonary disease and diseases contraindicating physical activity, no cancer, no psychiatric illness - additional inclusion for females: no pregnancy or breast-feeding - no specific diet - PAR-Q pass - body weight stable for the last 2 months and not on a weight-loss plan - no hypoglycemic, lipid-lowering, antihypertensive, psychiatric medications or medications known to affect body weight or energy expenditure |

**Table S2. Diet characteristics before (7-day record) and during the 12-week intervention** (Cipryan et al.. 2021)**.**

|  | **PRE** | | | | **DURING** | | | |
| --- | --- | --- | --- | --- | --- | --- | --- | --- |
|  | **HIIT** | **VLCHF** | **VLCHF+HIIT** | **Control** | **HIIT** | **VLCHF** | **VLCHF+HIIT** | **Control** |
| Energy (kJ) | 6900 (6000; 9100) | 7300 (6000; 8400) | 7900 (6300; 9100) | 7900 (6600; 10900) | 6800 (5300; 7900) | 5900 (4900; 6400) | 5900 (5100; 6500) | 7400 (5900; 10100) |
| Protein (g) | 70 (65; 86) | 63 (53; 86) | 75 (65; 88) | 79 (61; 88) | 67 (56; 76) | 74 (63; 86) | 74 (66; 89) | 76 (64; 85) |
| CHO (g) | 162 (140; 236) | 165 (154; 211) | 196 (146; 239) | 198 (175; 270) | 161 (122; 201) | 35 (28; 39) | 34 (25; 39) | 173 (150; 254) |
| Fat (g) | 67 (57; 99) | 68 (59; 86) | 81 (60; 95) | 78 (61; 106) | 68 (45; 87) | 104 (87; 118) | 101 (83; 118) | 68 (61; 94) |
| SFA (g) | 23 (17; 30) | 23 (18; 30) | 23 (18; 29) | 21 (18; 30) | 20 (14; 25) | 40 (31; 49) | 39 (31; 48) | 22 (17; 31) |
| Fiber (g) | 13 (10; 17) | 12 (10; 18) | 16 (13; 20) | 16 (11; 22) | 13 (9; 15) | 9 (7; 12) | 10 (8; 14) | 14 (11; 16) |

Legend: CHO – carbohydrates, SFA – saturated fatty acids.

Values are shown as median (lower and upper quartile).

**Table S3. 4-week biochemical analysis outcomes**

|  | **HIIT** | **VLCHF** | **VLCHF+HIIT** | **Control** |
| --- | --- | --- | --- | --- |
|  | ***M* (IQR)** | ***M* (IQR)** | ***M* (IQR)** | ***M* (IQR)** |
| Hemoglobin (g/l) | 134 (132; 140) | 141 (132; 150) | 137 (132; 142) | 137 (130; 148) |
| Hematocrit (%) | 0.394 (0.383; 0.416) | 0.411 (0.387; 0.439) | 0.404 (0.392; 0.42) | 0.398 (0.385; 0.422) |
| Erythrocytes (10^12^/l) | 4.43 (4.29; 4.64) | 4.62 (4.33; 5.26) | 4.56 (4.31; 4.89) | 4.59 (4.35; 4.83) |
| Thrombocytes (10^9^/l) | 238 (207; 287) | 232 (177; 269) | 220 (187; 263) | 238 (215; 285) |
| Leukocytes (10^9^/l) | 6 (5.3; 6.8) | 6.2 (5.4; 7.5) | 5.8 (5.1; 7.2) | 6.3 (5.3; 9.6) |
| HbA1c (mmol/mol) | 35.5 (32.5; 38) | 35 (33; 38) | 35 (32; 37.5) | 34 (32.8; 36.3) |
| Glucose (mmol/l) | 5.08 (4.87; 5.28) | 5.03 (4.73; 5.3) | 4.85 (4.54; 5.42) | 4.88 (4.7; 5.4) |
| Triglycerides (mmol/l) | 1.24 (0.98; 2.1) | 1.32 (0.9; 1.49) | 1.02 (0.83; 1.7) | 1.37 (0.9; 2.04) |
| Cholesterol (mmol/l) | 5.31 (4.29; 5.86) | 5.74 (4.49; 6.75) | 5.17 (4.28; 5.81) | 5.3 (4.93; 5.6) |
| HDL-C (mmol/l) | 1.3 (1.07; 1.47) | 1.19 (0.97; 1.45) | 1.21 (0.97; 1.63) | 1.23 (1.06; 1.47) |
| LDL-C (mmol/l) | 3.29 (2.54; 3.69) | 3.69 (2.79; 4.31) | 3.17 (2.36; 3.61) | 3.38 (2.85; 3.49) |
| Insulin (mU/l) | 8.3 (5.2; 13.6) | 7.5 (5.9; 10.5) | 7.1 (3.9; 11.9) | 10.1 (5.2; 17) |
| Leptin (ng/l) | 4.7 (2.9; 7.2) | 2.4 (1.1; 6.4) | 4.1 (1.3; 8.4) | 6 (3.1; 8) |
| Adiponectin (mg/l) | 26 (15; 45) | 39 (23; 60) | 44 (18; 58) | 52 (25; 89) |
| TG/HDL-C (-) | 0.94 (0.71; 1.46) | 1.18 (0.66; 1.52) | 0.82 (0.58; 1.49) | 1.02 (0.62; 1.86) |
| TyG index (-) | 3.25 (2.55; 5.25) | 3.14 (2.27; 4.01) | 2.86 (1.91; 4.13) | 3.4 (2.19; 5) |
| HOMA-IR (-) | 1.89 (1.21; 3.26) | 1.64 (1.23; 2.37) | 1.71 (0.79; 2.73) | 2.1 (1.07; 3.61) |
| Adpn/Lep (-) | 6.41 (3.93; 8.96) | 9.96 (5.33; 40.43) | 8.46 (4.6; 19.1) | 11.89 (3.82; 28.82) |

Legend*:* HbA1c – glycated hemoglobin, TG – triglycerides, HDL-C/LDL-C – high/low density lipoprotein, TyG index – triglyceride-glucose index, HOMA-IR – homeostatic model assessment of insulin resistance, Adpn/Lep – adiponectin-leptin index.

Data are the median (*M*) with interquartile range (IQR).

**Table S4. 12-week biochemical analysis outcomes**

|  | **HIIT** | **VLCHF** | **VLCHF+HIIT** | **Control** |
| --- | --- | --- | --- | --- |
|  | ***M* (IQR)** | ***M* (IQR)** | ***M* (IQR)** | ***M* (IQR)** |
| Hemoglobin (g/l) | 137 (131; 141) | 139 (132; 148) | 135 (128; 142) | 137 (130; 142) |
| Hematocrit (%) | 0.4 (0.384; 0.415) | 0.414 (0.384; 0.431) | 0.398 (0.378; 0.416) | 0.398 (0.382; 0.414) |
| Erythrocytes (10^12^/l) | 4.47 (4.28; 4.73) | 4.58 (4.26; 5.08) | 4.5 (4.2; 4.86) | 4.56 (4.31; 4.72) |
| Thrombocytes (10^9^/l) | 248 (214; 297) | 237 (197; 270) | 210 (179; 260) | 241 (206; 280) |
| Leukocytes (10^9^/l) | 6.3 (5.4; 7) | 6.4 (5.8; 7.4) | 5.5 (4.3; 6.4) | 6 (5.3; 7.4) |
| HbA1c (mmol/mol) | 36.5 (29.8; 40) | 33 (31.5; 37) | 34 (30.5; 37) | 36 (33; 36) |
| Glucose (mmol/l) | 4.93 (4.75; 5.45) | 5.13 (4.73; 5.37) | 5.02 (4.47; 5.48) | 4.81 (4.44; 5.15) |
| Triglycerides (mmol/l) | 1.15 (0.73; 1.68) | 0.99 (0.78; 1.62) | 0.78 (0.72; 1.15) | 1.39 (0.84; 2.54) |
| Cholesterol (mmol/l) | 4.78 (4.38; 5.72) | 5.61 (4.9; 6.65) | 5.3 (4.6; 6.16) | 5.25 (4.4; 6.05) |
| HDL-C (mmol/l) | 1.25 (1.07; 1.49) | 1.28 (1.05; 1.49) | 1.36 (1.12; 1.63) | 1.19 (1.05; 1.5) |
| LDL-C (mmol/l) | 2.96 (2.64; 3.54) | 3.72 (2.93; 4.45) | 3.32 (2.73; 3.77) | 3.15 (2.73; 3.65) |
| Insulin (mU/l) | 8.1 (4.7; 12.8) | 6.1 (3.9; 10.2) | 5.3 (4; 10.5) | 9.6 (6.7; 13.2) |
| Leptin (ng/l) | 5.2 (1.9; 7.6) | 3.2 (1.7; 5.7) | 4 (1.4; 8.6) | 8.7 (6.3; 16.8) |
| Adiponectin (mg/l) | 25 (14; 47) | 57 (26; 84) | 36 (23; 55) | 38 (28; 55) |
| TG/HDL-C (-) | 0.78 (0.6; 1.48) | 0.85 (0.51; 1.59) | 0.61 (0.47; 1.12) | 1.15 (0.53; 2.36) |
| TyG index (-) | 3.13 (1.62; 4.23) | 2.5 (1.74; 3.83) | 2.03 (1.77; 3.42) | 3.29 (1.99; 6.4) |
| HOMA-IR (-) | 1.95 (0.94; 3.24) | 1.31 (0.83; 2.24) | 1.17 (0.88; 2.48) | 1.92 (1.27; 2.7) |
| Adpn/Lep (-) | 6.44 (4.52; 9.31) | 18.87 (7.31; 40.49) | 11.9 (6.34; 33.85) | 4.95 (2.51; 6.31) |

Legend*:* HbA1c – glycated haemoglobin, TG – triglycerides, HDL-C/LDL-C – high/low density lipoprotein, TyG index – triglyceride-glucose index, HOMA-IR – homeostatic model assessment of insulin resistance, Adpn/Lep – adiponectin-leptin index.

Data are the median (*M*) with interquartile range (IQR).

**Table S5. Biochemical variables differences after 4 weeks**

|  | **HIIT** | **VLCHF** | **VLCHF+HIIT** | **Control** | **Between-group diff.**  **(*p*-value)** |
| --- | --- | --- | --- | --- | --- |
|  | **∆*M* (95% CI)** | **∆*M* (95% CI)** | **∆*M* (95% CI)** | **∆*M* (95% CI)** |  |
| Hemoglobin (g/l) | -2.5 (-3.5; 1.0) | 1.0 (-1.0; 2.5) | -1.0 (-3.5; 1.0) | -3.0 (-4.5; 0.5) | 0.270 |
| Hematocrit (%) | -0.01 (-0.01; 0.00) | 0.00 (0.00; 0.01) | 0.00 (-0.01; 0.00) | -0.01 (-0.02; 0.00)* | 0.058 |
| Erythrocytes (10^12^/l) | -0.07 (-0.11; 0.02) | 0.04 (-0.01; 0.13) | 0.00 (-0.11; 0.06) | -0.05 (-0.18; -0.01)* | 0.030^a^ |
| Thrombocytes (10^9^/l) | -5.0 (-13.0; 2.5) | -1.0 (-15.0; 13.5) | -20.0 (-26.0; -4.0)* | 1.0 (-8.5; 5.5) | 0.133 |
| Leukocytes (10^9^/l) | -0.35 (-0.75; 0.05) | 0.30 (-0.25; 0.90) | -0.50 (-0.85; 0.10) | -0.40 (-0.90; 0.40) | 0.139 |
| HbA1c (mmol/mol) | 0.0 (-2.0; 0.5) | 0.0 (-2.5; 1.5) | 0.0 (-2.0; 1.5) | -1.0 (-2.0; 0.0) | 0.705 |
| Glucose (mmol/l) | 0.02 (-0.26; 0.20) | -0.31 (-0.71; -0.06)* | -0.26 (-0.48; 0.09) | -0.04 (-0.42; 0.22) | 0.404 |
| Triglycerides (mmol/l) | 0.17 (-0.20; 0.74) | -0.05 (-0.58; 0.20) | -0.03 (-0.43; 0.11) | -0.10 (-0.48; 0.25) | 0.497 |
| Cholesterol (mmol/l) | -0.11 (-0.38; 0.25) | -0.01 (-0.46; 0.39) | -0.10 (-0.57; 0.23) | -0.16 (-0.56; 0.07) | 0.791 |
| HDL-C (mmol/l) | -0.07 (-0.13; 0.03) | -0.07 (-0.19; 0.01) | -0.08 (-0.15; 0.07) | 0.03 (-0.17; 0.07) | 0.830 |
| LDL-C (mmol/l) | -0.03 (-0.37; 0.15) | 0.06 (-0.25; 0.36) | 0.05 (-0.25; 0.23) | -0.16 (-0.42; -0.02)* | 0.420 |
| Insulin (mU/l) | 1.76 (-0.79; 5.39) | -1.69 (-3.57; -0.81)* | -1.52 (-3.68; 0.14) | -1.43 (-3.21; 3.12) | 0.041^b^ |
| Leptin (ng/l) | -0.08 (-1.23; 0.52) | -3.24 (-5.60; -2.82)** | -2.89 (-4.64; -1.94)** | 0.38 (-0.59; 1.05) | < 0.001^c^ |
| Adiponectin (mg/l) | 1.75 (-6.60; 10.13) | 2.00 (-9.18; 4.39) | -1.44 (-10.93; 4.98) | 17.66 (-0.73; 35.88) | 0.127 |
| TG/HDL-C (-) | 0.13 (-0.08; 0.65) | -0.05 (-0.30; 0.19) | -0.09 (-0.34; 0.09) | 0.07 (-0.45; 0.30) | 0.339 |
| TyG index (-) | 0.59 (-0.60; 1.96) | -0.18 (-1.93; 0.28) | -0.24 (-1.14; 0.23) | -0.37 (-1.37; 0.73) | 0.468 |
| HOMA-IR (-) | 0.34 (-0.22; 1.19) | -0.43 (-1.17; -0.20)* | -0.37 (-1.03; 0.10) | -0.47 (-0.83; 0.98) | 0.047^b^ |
| Adpn/Lep (-) | 0.72 (-1.26; 1.42) | 4.00 (3.04; 35.79)** | 2.32 (1.15; 7.73)* | 1.01 (-0.55; 9.21) | 0.003^b^ |

Legend*:* HbA1c – glycated haemoglobin, TG – triglycerides, HDL-C/LDL-C – high/low density lipoprotein, TyG index – triglyceride-glucose index, HOMA-IR – homeostatic model assessment of insulin resistance, Adpn/Lep – adiponectin-leptin index.

Data are the median differences (∆*M*) between baseline minus 4-week measures with 95% confidence intervals (CI).

Two-tailed Wilcoxon signed-rank test: * significant differences (p<0.05) for baseline vs. 4-week; ** significant differences (p<0.001) for baseline vs. 4-week.

Kruskal-Wallis test for the between-group differences. Post-hoc analysis (homogenous subgroups): ^a^ – (HIIT, Control and VLCHF+HIIT); (VLCHF, VLCHF+HIIT and HIIT), ^b^ – (HIIT, Control and VLCHF+HIIT)(VLCHF, VLCHF+HIIT and Control), ^c^ – (HIIT and Control); (VLCHF and VLCHF+HIIT),.

**Table S6. Number of values (% of all values in the group) under a detection level of the assay**

Variables excluded from further statistical analysis.

|  | **HIIT**  (N=22) | **VLCHF**  (N=25) | **VLCHF+HIIT**  (N=25) | **Control**  (N=19) |
| --- | --- | --- | --- | --- |
| **Baseline/12 weeks** |  |  |  |  |
| Lipoprotein (a) | 9/8 (41/36 %) | 14/15 (56/60 %) | 15/16 (60/64 %) | 12/13 (63/68 %) |
| TNF-α | 3/5 (14/23 %) | 2/6 (8/24 %) | 6/10 (24/40 %) | 5/7 (26/37 %) |
| hs-IL-6 | 7/9 (32/41 %) | 4/12 (16/48 %) | 8/10 (32/40 %) | 7/4 (37/21 %) |
| IL-1RA | 17/16 (77/73 %) | 13/15 (52/60 %) | 21/20 (84/80 %) | 10/13 (53/68 %) |
| IL-1β | 21/21 (96/96 %) | 25/25 (100/100 %) | 24/22 (96/88 %) | 16/18 (84/95 %) |
| IL-10 | 21/20 (96/91 %) | 24/24 (96/96 %) | 24/22 (96/88 %) | 16/18 (84/95 %) |

Legend: absolute number at baseline/12 weeks with percentage of total number of participants in the group.

References:

Cipryan. L.. Dostal. T.. Litschmannova. M.. Hofmann. P.. Maffetone. P. B.. & Laursen. P. B. (2021). Effects of a Very Low-Carbohydrate High-Fat Diet and High-Intensity Interval Training on Visceral Fat Deposition and Cardiorespiratory Fitness in Overfat Individuals: A Randomized Controlled Clinical Trial. *Frontiers in Nutrition*. *8*(December). 1–13. https://doi.org/10.3389/fnut.2021.785694
